# Recent Advances in Nanozyme-Based Sensing Technology for Antioxidant Detection

**DOI:** 10.3390/s24206616

**Published:** 2024-10-14

**Authors:** Xin Cao, Tianyu Liu, Xianping Wang, Yueting Yu, Yangguang Li, Lu Zhang

**Affiliations:** 1 School of Pharmaceutical Sciences and Institute of Materia Medica, Xinjiang University, Urumqi 830017, China; caoxin@stu.xju.edu.cn (X.C.); liutianyu@stu.xju.edu.cn (T.L.); wxp@stu.xju.edu.cn (X.W.); yuyueting@stu.xju.edu.cn (Y.Y.); 2 College of Intelligent Manufacturing and Modern Industry, Xinjiang University, Urumqi 830017, China; 3 Bingtuan Energy Development Institute, Shihezi University, Shihezi 832000, China

**Keywords:** antioxidant, nanozyme, colorimetric detection, fluorescence detection, electrochemical technology

## Abstract

Antioxidants are substances that have the ability to resist or delay oxidative damage. Antioxidants can be used not only for the diagnosis and prevention of vascular diseases, but also for food preservation and industrial production. However, due to the excessive use of antioxidants, it can cause environmental pollution and endanger human health. It can be seen that the development of antioxidant detection technology is important for environment/health maintenance. It is found that traditional detection methods, including high performance liquid chromatography, gas chromatography, etc., have shortcomings such as cumbersome operation and high cost. In contrast, the nanozyme-based detection method features advantages of low cost, simple operation, and rapidity, which has been widely used in the detection of various substances such as glucose and antioxidants. This article focuses on the latest research progress of nanozymes for antioxidant detection. Nanozymes for antioxidant detection are classified according to enzyme-like types. Different types of nanozyme-based sensing strategies and detection devices are summarized. Based on the summary and analysis, one can find that the development of commercial nanozyme-based devices for the practical detection of antioxidants is still challenging. Some emerging technologies (such as artificial intelligence) should be fully utilized to improve the detection sensitivity and accuracy. This article aims to emphasize the application prospects of nanozymes in antioxidant detection and to provide new ideas and inspiration for the development of detection methods.

## 1. Introduction

Antioxidant is defined as any substance that has the ability to prevent or mitigate oxidative damage from free radicals and is widely found in foods, cosmetics, and pharmaceuticals. Antioxidants come from a rich variety of sources, including plants and animals. Antioxidants are common in nature, with polyphenols, carotenoids, and vitamins being the major antioxidant compounds. These antioxidants have been shown to have the ability to prevent oxidative damage caused by free radicals and protect the human body from oxidative stress. Moreover, they can be used in the treatment of many types of diseases, such as cardiovascular [1,2,3], diabetes [4,5], Parkinson [6,7,8], and cancer [9,10]. At the same time, antioxidants serve to maintain the freshness of foodstuffs by preventing or delaying the oxidation process. Vitamin E is frequently employed in the storage of vegetable oils to prevent the oxidation of their oils. Additionally, ascorbic acid (AA) is often used to prevent the oxidation of dried fruits and nuts in order to preserve their color and nutritional value. The proper use of antioxidants not only helps to extend the storage and marketing period of food products, bringing great economic benefit to both producers and consumers, but also ensures the food safety of consumers. Furthermore, thanks to their low cost and ease of production, antioxidants are often used in the production of daily necessities such as plastics and rubber to prevent oxidation and corrosion [11]. On the other hand, due to the excessive use of antioxidants, they are ubiquitous in natural environments such as the ocean and atmosphere, resulting in accumulation within organisms. There is also a growing concern regarding the potential adverse effects of antioxidants on biological populations. Therefore, the detection of antioxidants is crucial for human health, industrial production, food preservation, and environmental protection.

In recent years, many methods have been used to detect antioxidant substances, including high performance liquid chromatography [12,13,14,15], gas chromatography [16,17,18,19], 2,2-Diphenyl-1-picrylhydrazyl free radical scavenging assay [20,21,22,23,24], 2,2′-Azino-bis(3-ethylbenzothiazoline-6-sulfonic acid) free radical scavenging assay [25,26,27,28], protein oxidative damage assay [29], fluorescence recovery after photobleaching [30,31] and oxygen radical absorbance capacity [32,33], enzyme assay [34,35], etc. Using enzymes to detect antioxidant properties is an effective method. Based on the source of enzymes, they can be classified into natural and artificial enzymes. Natural enzymes have a wide range of prospects and applications. However, they have disadvantages, such as high preparation costs and poor stability [36]. In order to overcome these drawbacks, researchers have begun to study nanozymes, artificial enzymes based on nanomaterials [37]. Over the past decades, nanozymes have emerged as an interdisciplinary field in nanotechnology, catalysis, and biomedicine [38,39]. Nanozymes are nanomaterials with catalytic activity similar to that of natural enzymes. They exhibit excellent biological enzyme activity. Due to their distinctive structural features, nanozymes display unique physicochemical properties at the nanoscale. These properties enable them to perform functions analogous to natural enzymes, including hydrolysis, redox reactions and cleavage. Nanozymes are prepared by a variety of methods, subdivided into three main categories: chemical synthesis, biosynthesis, and self-assembly. Chemical synthesis method, is usually considered as the simplest preparation method, includes sol-gel method [40,41], precipitation method [42,43,44,45,46], solvothermal synthesis [47,48,49,50], hydrothermal method [51,52,53,54,55,56,57,58,59], etc. It has the advantages of simple operation, low requirements, and good controllability. The large-scale preparation of nanozymes can be easily achieved by using suitable chemical synthesis methods. Compared with natural enzymes, nanozymes have the advantage of high stability, low production costs, high activity, and are easier to produce and recycle [60]. With the booming development of biotechnology, nanotechnology, and catalysis technology, nanozymes are gradually used instead of natural enzymes in industrial production [36], pharmaceutical field [61,62,63,64,65], environmental monitoring [66], energy development [67], food testing [68], and ecological protection [69]. In daily life, nanozymes are often used to detect the antioxidant properties of substances efficiently and rapidly.

As of 2024, many works on nanozymes have been published. Humans gradually master more detection technologies using nanozymes. At present, there are some reviews on the application of nanozymes in specific fields, such as food detection [70,71,72], environmental monitoring [73,74,75,76], medical diagnosis [77,78], and so on. However, comprehensive classification or summary of the applications of nanozymes and nanozyme-based derivative devices in terms of antioxidant properties is lacking. The purpose of this work is to systematically review the application of novel nanozymes for antioxidant detection. In this work, various detection devices and sensing strategies based on nanozymes are described in detail. At the same time, the basic principles and advantages of detection devices, such as sensor arrays, in terms of oxidation resistance are reviewed. We believe that this paper will not only facilitate an understanding of the application of nanozymes in antioxidant detection but also encourage further exploration of the potential applications of nanomaterials in the field of biosensing.

## 2. Principle of Nanozymes in Antioxidant Detection

**Figure 1:**
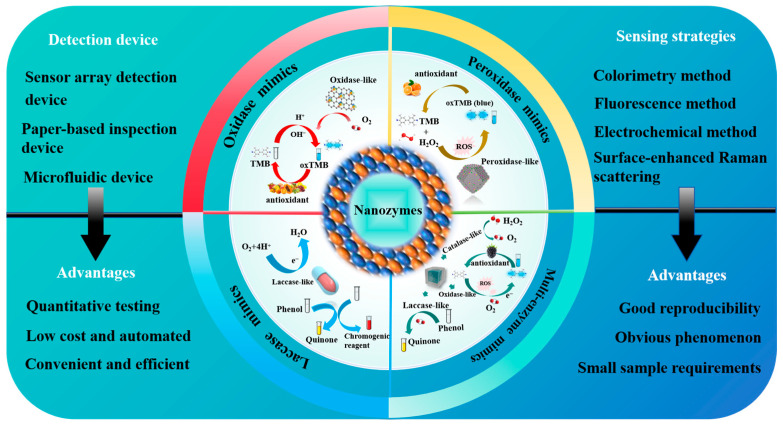
Classification of catalytic activity of nanozymes for oxidation assays.

Nanozymes range in size from 1 to 100 nm [79]. Two key factors for evaluating their catalytic performance are catalytic activity and specificity [80]. The catalytic activity of a nanozyme is affected by the composition, spatial structure, substrate characteristics, interface effect, morphology, and environment. The catalytic specificity of a nanozyme is divided into four types: reaction specificity, substrate specificity, regional specificity, and stereospecificity [80], which are affected by surface properties, substance composition, substrate structure, kinetic factors, steric hindrance, and other factors. Nanozymes are a type of nanomaterial that can imitate the catalytic activity of natural enzymes [81,82,83,84] and can exhibit extremely similar kinetic behavior to natural enzymes [60]. Therefore, they are promising candidates as new artificial enzymes [85,86,87]. Based on their catalytic properties, nanozymes are classified into several categories: peroxidases (POD), oxidases (OXD), catalases, superoxide dismutase, laccases, etc. [79]. Among these, POD nanozymes, OXD nanozymes, and laccase are often used in antioxidant assays (see Figure 1).

### 2.1. POD Nanozymes for Antioxidant Detection

**Figure 2:**
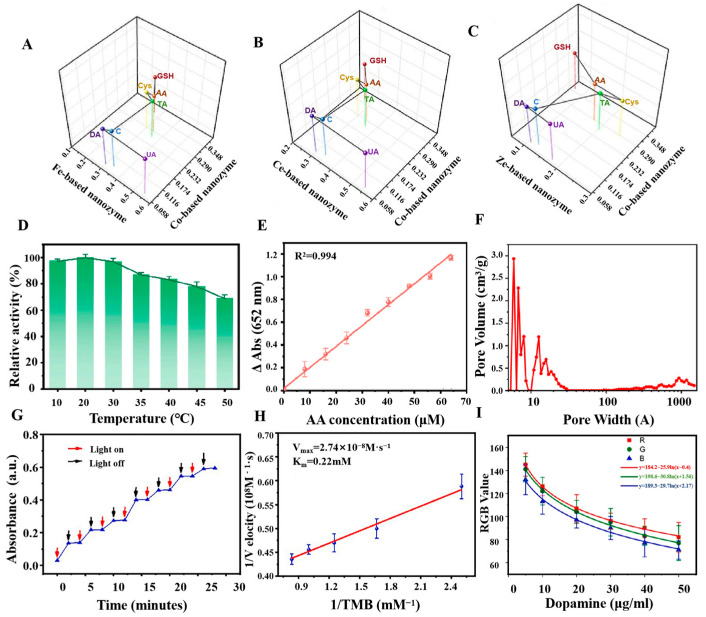
(**A**–**C**) Center of mass diagram for differentiation of seven antioxidants [89]. (**D**) Relative catalytic activity of the catalysts at different temperatures [90]. (**E**) Detection of calibration curves corresponding to AA [90]. (**F**) Pore width distribution of NPS-C nano-enzymes [99]. (**G**) Photo-controllable mimic OXD activity of CDs [94]. (**H**) Lineweaver-Burk plot of the light-responsive OXD-like activity of CDs [94]. (**I**) Detection of dopamine (DA) concentration [69].

POD-like nanozyme is a class of oxidoreductases that has similar biological activity to natural POD and is able to participate in many types of redox reactions [88]. The specificity of POD nanozymes is stronger than that of other types of enzymes, and they are mainly involved in catalyzing the redox reaction between H_2_O_2_ and the substrate, giving them high specificity in the antioxidant detection reactions containing H_2_O_2_. POD nanozyme is frequently employed as substitutes for natural POD in assay and fermentation applications, where they play a pivotal role, particularly in the detection of antioxidant properties of substances (see Table 1). Furthermore, in comparison to natural POD, POD nanozyme exhibits enhanced specificity and selectivity, as well as the capacity to effectively catalyze intricate chemical reactions. Qin et al. have developed a metal-based nanozyme with a three-dimensional network structure using four metals (Co, Fe, Cu, Zn) [89], which led to the creation of a simulated enzyme with exceptional POD properties. In Figure 2A–C, a sensor array based on three metal-based POD nanozymes can be used to sensitively detect a variety of antioxidants, including seven antioxidants at concentrations as low as 10 nmol/L. The consumption of foods with high total antioxidant capacity (TAC) can reduce the incidence of many diseases. Therefore, the establishment of a platform for the testing of TAC is particularly important. Dan et al. have established a TAC detection platform using 3,3′,5,5′-Tetramethylbenzidine (TMB) by synthesizing the high POD nanozyme Bi_0.3_Fe_1.7_MoO_6_ under H_2_O_2_ conditions [90]. The Bi_0.3_Fe_1.7_MoO_6_ nanozyme displayed varying relative catalytic activities at different temperatures, exhibiting high catalytic activity within the range of 10 °C to 30 °C. Consequently, this nanozyme can be applied at room temperature (see Figure 2D). At the same time, due to the high affinity of Bi_0.3_Fe_1.7_MoO_6_ for H_2_O_2_ and TMB, it achieved a detection limit as low as 0.77 μmol/L and a linear range of 8 to 64 μmol/L, with an excellent correlation coefficient (*R*^2^ = 0.994). Figure 2E demonstrates that this assay platform is suitable for the detection of AA, exhibiting a minimal detection level and a satisfactory linear range (8–64 μmol/L) with an excellent correlation coefficient (*R*^2^ = 0.994). Wang et al. have successfully developed a nitrogen, phosphorus, and sulfur co-doped carbon nanoparticle enzyme (NPS-C) through the process of high-temperature pyrolysis. NPS-C nanozymes exhibited superior specific activity and enhanced substrate binding affinity compared to NP-C, NS-C, and N-C nanozymes. Furthermore, the TMB-H_2_O_2_ system can be efficiently catalyzed by doping with NPS-C nanozyme, which generated reactive oxygen intermediates and superoxide anion radicals (•O^2−^) with high oxidative capacity. This promoted the electron-loss oxidation reaction process of TMB. Consequently, NPS-C was capable of ascertaining the antioxidant characteristics of compounds such as AA, l-cystine (L-Cys), and glutathione (GSH) through the indirect detection of TAC. Figure 2F illustrates that NPS-C exhibits an excess of microporous and mesoporous structures, along with a substantial specific surface area, which provides an adequate number of binding sites for the antioxidant assay process.

### 2.2. OXD Nanozymes for Antioxidant Detection

The principal distinction between OXD-like nanozymes and nanozymes such as POD is that OXD possess a more expansive range of substrates, and are capable of participating in a multitude of redox reactions. They are predominantly involved in reactions involving oxygen as the ultimate electron acceptor. OXD combines the unique properties of nanomaterials and the efficient catalytic ability of biological enzymes, with the advantages of low production cost, high stability, and easy regulation of activity. In comparison to conventional natural enzymes, OXD nanozymes show a wide range of application potential in the fields of biology, medicine, analytical sensing, and environmental treatment. In contrast to other forms of nanozymes, the conditions for the utilization of OXD were particularly rigorous, necessitating precise pH and temperature control. The data (Table 2) show that OXD nanozymes exhibit a markedly higher sensitivity than other types of enzymes [91,92,93], enabling the detection of lower concentrations of antioxidants. In the context of antioxidant assays, OXD nanozymes typically demonstrate antioxidant activity by catalyzing a specific substrate. To detect antioxidants in fruits, Li et al. proposed a practical strategy for TAC assessment [94]. An active CD nanozyme with OXD-like activity was developed by utilizing precursors with abundant electron-donating and electron-drawing groups. Its activity was controlled by light, as illustrated in Figure 2G. As shown in Figure 2H, CDs with OXD-like activity display favorable compatibility with TMB, with an affinity as low as 0.22 mmol/L. Li et al. have constructed a sensor through a coupling reaction between CDs and TMB [94], achieving the successful detection of antioxidants and TAC in fruits with high sensitivity and accuracy. Similarly, Ni et al. have established a simple, sensitive, and efficient antioxidant checking method using photoresponsive BSA-AuNCs with OXD-like activity [95]. The detection principle is that antioxidants can inhibit the photoactivated OXD simulation activity of BSA-AuNCs [95], thus achieving the purpose of inhibiting the formation of fluorescent thiopigments. At the same time, relevant experiments showed that the OXD had higher sensitivity and accuracy in some environments. For example, in an unstable H_2_O_2_ environment, the POD-catalyzed reaction may face problems with the reproducibility of the sensed signal, which can affect the accuracy of the detection. Wang et al. have constructed a Pt-Ni NPs-like OXD nanozymes, consisting of a Pt-rich shell and a Ni-rich nucleus, which solved the challenge of reproducibility of POD under unstable H_2_O_2_ conditions [96]. This OXD nanozyme was successfully used for the detection of many types of antioxidant substances, such as small bioactive molecules, nanomaterials, cells, etc., which provided a new solution for the preparation of OXD nanozymes with high activity.

### 2.3. Laccase-like Nanozymes for Antioxidant Detection

The high cost, short storage life, and poor reusability of natural lacquers have hindered their widespread use in industry [97]. However, the simulated laccase nanozyme solves these limitations, and is widely used in the fields of sensors, environmental monitoring, medical diagnosis, and treatment. In antioxidant capacity assays, laccase-like nanozymes are demonstrably more effective than other types of enzymes in terms of assay flexibility. Laccase-like nanozymes can catalyze the reaction of polyphenols with hydrogen peroxide to produce quinones or free radical products, which can be quantified by means of colorimetry, electrochemistry, etc. As shown in Table 3, the concentration and nature of the oxidation products produced by this process can indirectly reflect the phenolic content and thus assess the antioxidant capacity of the samples. For example, Huang et al. have prepared an AMP-Cu nanozyme with laccase-like catalytic properties, which catalyzed the oxidation of phenolic compounds. Furthermore, the researchers have employed 2,4-dinitrophenol (2,4-DP) as the substrate and 4-Aminophenol (4-AP) as the color developer to quantify the absorbance of the products at 510 nm, thereby detecting phenolic compounds and evaluating the antioxidant capacity of various fruit juices [98]. This approach enabled the sensitive and cost-effective detection of polyphenols and antioxidant properties. Similarly, Wang et al. have been used an I-Cu nanozyme with high laccase activity [69], which can generate different colored products by oxidizing phenolic compounds. After completing the color development reaction, the antioxidant capacity of the sample can be assessed using the smartphone, combined with the accurate measurement of the antioxidant content in the sample (see Figure 2I). The efficacy of laccase-like nanozymes in antioxidant assays was contingent not only upon their simulated catalytic activity but also upon a multitude of environmental factors, including pH, temperature, substrate concentration, and substrate species. Consequently, the potential of nanozymes in determining antioxidant capacity can be further elucidated through the control and the optimization of these conditions.

### 2.4. Multi-Enzyme Nanozymes for Antioxidant Detection

**Figure 3:**
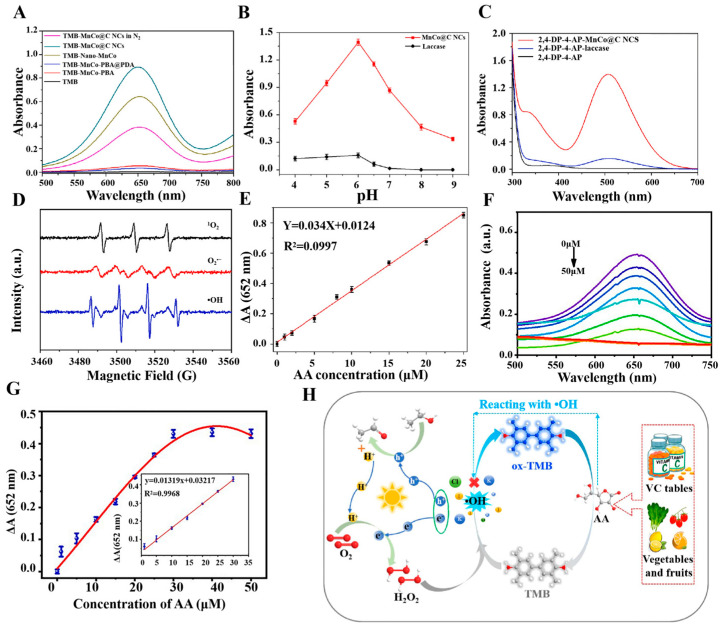
(**A**) UV–Vis absorption spectra in diverse reaction systems in different reaction systems [106]. (**B**) Effect of pH on the catalytic activity of MnCo@C NCs and laccase [101]. (**C**) UV-visible of 2,4-DP-4-AP, 2,4-DP-4-AP-laccase, and 2,4-DP-4-AP-MnCo@C NCs absorption spectra [101]. (**D**) Electron Spin Resonance signals detected by ^1^O_2_, O_2_^•−^ and •OH [101]. (**E**) Linear fit plot of absorbance versus AA concentration [101]. (**F**) UV–Vis absorption spectra of different concentrations of AA(0, 1, 5, 10, 15, 20, 25, 30, 40, and 50 μmol/L) [94]. (**G**) Corresponding calibration curves and linear relationships [94]. (**H**) TAC assessment of substances by modified carbon nitride nanozymes [109].

A multi-enzyme nanozyme, is nanozyme with two or more enzyme-like activities [100]. Table 4 shows that, unlike other types of nanozymes, multi-enzyme nanozyme has a wide range of catalytic abilities and potential applications, gradually taking their place in the field of antioxidant assays. The multi-enzymatic nanozyme, such as MnCo@C NCs nanozyme, has highly efficient catalytic properties and multiple enzyme mimetic activities of OXD, laccases, and POD. Meanwhile, MnCo@C NCs nanozyme also has the advantages of high stability and reproducibility, and can maintain high catalytic activity under harsh environments. Zhu et al. have employed MnCo@C NCs nanozymes to detect the antioxidant properties of a range of samples, including vegetables, fruits, beverages, and human serum [101]. The assay process achieved a synergistic effect of multiple enzyme activities. The MnCo@C NCs nanozymes displayed POD activity, which enabled the decomposition of hydrogen peroxide within the original reaction system and the prevention of its oxidation of vitamin C. This ensured that the competitive reaction between vitamin C and reactive oxygen species proceeded in a normal and smooth manner. The MnCo@C NCs nanozymes displayed OXD activity, oxidizing the colorless TMB substrate to produce the blue ox-TMB product in a synergistic detection process (see Figure 3A). The nanozyme produced by MnCo@C exhibited higher levels of activity than natural laccase across a pH range of 4 to 9. However, both enzymes appear to display optimal performance at a pH level of approximately 6, as shown in Figure 3B. In the synergistic process, laccase activity primarily served as a catalyst for the oxidation of phenolic compounds, with high sensitivity reflected in Figure 3C. The remarkable activity of the MnCo@C NCs nanozymes was attributed to the collective action of multiple reactive oxygen species. By enabling sensitive detection of vitamin C and indirectly assessing the TAC of the samples, a robust foundation is established for the use of multienzyme nanozymes in assay applications (see Figure 3D). The absorbance difference obtained during the detection of AA using MnCo@C NCs nanozymes demonstrated an excellent linear relationship with AA concentration, with a limit of detection (LOD) of 0.29 μmol/L [101], as illustrated in Figure 3E. The variety of enzyme activities made multi-enzymes a new type of multifunctional catalyst that not only played a key role in the detection of antioxidants, but also allowed other enzyme activities to be used for the detection and catalysis of other substances. Liu and colleagues have synthesized a bimetallic oxide Co_1.5_Mn_1.5_O_4_ through a chemical process [102]. This material displayed four distinct enzyme mimetic activities, namely POD activity, OXD activity, catalase activity and laccase activity. The laccase and OXD activities of Co_1.5_Mn_1.5_O_4_ nanozyme were employed for the detection of catechol (CC) and hydroquinone (HQ), respectively, while the remaining two enzyme activities could be used for the detection or catalysis of other substances. The detection of the antioxidant substance CC depended on the laccase activity. Laccase was employed to oxidate CC and form o-benzoquinone, which resulted in an increase in the absorbance of the reaction solution, thus enabling the quantitative detection of CC. The OXD enzyme activity of Co_1.5_Mn_1.5_O_4_ nanozyme can form an assay system with TMB, which was oxidated to ox-TMB. Meanwhile, HQ reduced ox-TMB, resulting in a decrease in the absorbance. As a result, the decrease in absorbance allowed for the quantitative determination of HQ. The advent of multi-enzymatic nanozyme heralds a new era of nanozyme research, offering a plethora of novel concepts for the advancement of nanozyme in the area of immediate detection technology in food regulation, environmental monitoring, and human health.

## 3. Application of Nanozymes in Antioxidant Detection

### 3.1. Nanozymes-Based Densing Dethods for Dntioxidant Detection

Nanozyme, as an emerging biocatalyst, demonstrates distinctive advantages in the detection of antioxidant capacity. A variety of methods have been developed to determine antioxidant capacity, including colorimetry [93,103,104,105], fluorimetry [96], and electrochemical methods [106].

#### 3.1.1. Colorimetric Methods for Antioxidants Determination

**Figure 4:**
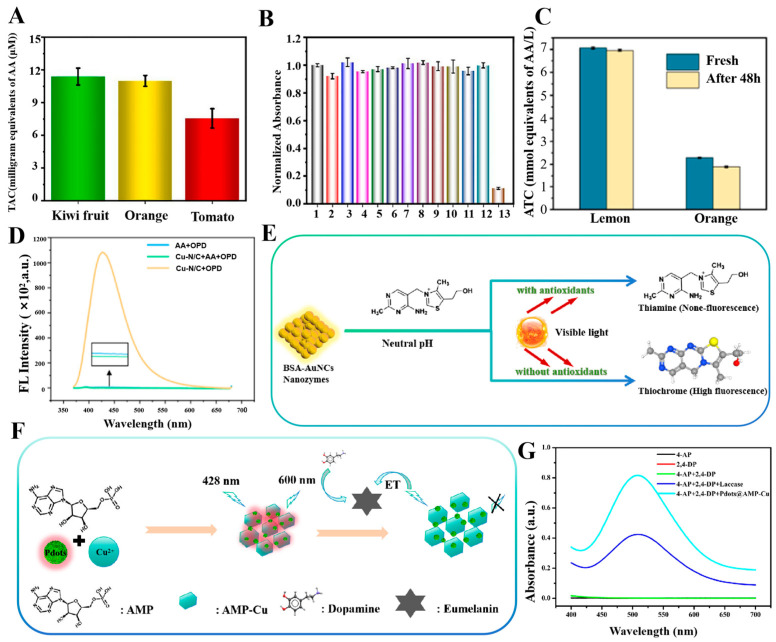
(**A**) Histograms of TAC of the three juices [94]. (**B**) Selectivity of the cd-coupled TMB system for AA detection in the presence of multiple metal ions and amino acids [94]. (**C**) TAC changes in oranges and lemons [110]. (**D**) Fluorescence signals of different reaction systems [112]. (**E**) Detection of antioxidants using the photoresponsiveness of BSA-AuNCs. (**F**) Schematic diagram of the Pdots@AMP-Cu-based DA detection strategy. (**G**) UV-visible absorption curves of different reaction solutions in MES buffer solution at pH = 7 [115].

In colorimetry assays, the nanozymes catalyze the colorimetric substrates (e.g., TMB and ABTS), which produce a colorimetric output signal. Due to its simplicity, convenience, speed, and economy, colorimetry is considered as the most common sensing mode and is often used for on-site testing of antioxidant capacity [107,108]. Li et al. have constructed a multifunctional colorimetric sensing platform by synthesizing modified carbon nitride nano-enzymes. For the first time, the self-cascade photocatalytic H_2_O_2_ production strategy was successfully applied to AA detection and TAC assessment, which proved the feasibility of this strategy in detecting antioxidant systems, as illustrated in Figure 3H. The chromogenic substrate TMB was employed in the experiments and the oxidation led to the formation of blue OX-TMB, exhibiting a characteristic absorption peak at a wavelength of 652 nm [109]. Li et al. have developed a novel light-responsive carbon dot with OXD-like activity that can catalyze an oxidation reaction to change the color of the color-developing substrate TMB under light stimulation. The characteristic absorption peak was observed at a wavelength of 652 nm by UV–Vis spectroscopy. Furthermore, the wavelength conditions of AA concentration and the change in absorbance of OX-TMB exhibited an excellent linear relationship, demonstrating the high sensitivity of the detection system, as depicted in Figure 3F,G. The TAC of three fruits— kiwifruit, orange, and tomato—was successfully detected by this colorimetric sensing with high accuracy and excellent selectivity, as shown in Figure 4A,B. This light-responsive property offers new possibilities for the modulation of oxidative enzyme activities, with enhanced environmental compatibility and biocompatibility [94]. Nitrogen, phosphorus, and sulfur co-doped carbon nanozyme (NPS-C) has been designed by Wang et al. through a one-step high-temperature calcination process. Compared with single-doped nanozymes, NPS-C exhibited enhanced POD-like activity and substrate binding affinity. It rapidly activated the oxidation substrate H_2_O_2_ within 5 min, which in turn promoted the oxidation reaction of the color-developing substrate TMB. The oxidation product showed an enhanced absorption signal in the UV–Vis absorption spectrum at 652 nm. The method exhibited excellent selectivity and resilience to interference in authentic samples, accurately quantifying AA and TAC in commercial beverages in the presence of interference from metal ions, amino acids, and small molecules [99]. Similarly, Wang et al. have prepared platinum-nickel nano-case rich in platinum shell and nickel core through a one-step high-temperature reduction strategy, resulting in unique structural characteristics. In contrast to POD-like nanozymes that depend on unstable hydrogen peroxide, Pt-NiNPs were capable of activating molecular oxygen and oxidizing the chromogenic substrate TMB in the absence of H₂O₂, thus producing an oxidate product with a distinctive absorption peak at 652 nm. This process avoided the challenge in reproducibility associated with the use of unstable H₂O₂. Platinum-nickel nanozyme was used to develop a bioassay platform for colorimetric detection of TAC. The antioxidant capacity of four small molecules—AA, glutathione, Cys, and 6-hydroxy-2,5,7, 8-tetramethylchromo-2-carboxylic acid—and three cells—Hela cells, human umbilical vein endothelial cells, and senescent HUVECs—was successfully detected [96]. Ni et al. have successfully prepared CuBi (copper-bismuth) bimetallic aerogel nanozymes with a porous structure and interlocked pores by a one-step reduction strategy. AA was employed as a representative antioxidant model to develop a colorimetric method for the detection of TAC. In the experiments, the CuBi bimetallic aerogel catalyzed the production of •OH from H_2_O_2_ and oxidated the colorless TMB, resulting in a blue product with an absorption peak at 652 nm. Three antioxidants—AA, glutathione, and Cys—were quantified, and the TAC of two different types of vitamin tablets, two types of fruits, and six beverages was accurately assessed, opening new research directions in the field of application of metal aerogels [110]. This method can be used in the field of food storage, as demonstrated in Figure 4C, which shows that lemon and orange exhibit a gradual decrease in storage.

#### 3.1.2. Fluorescence Method for Antioxidants Determination

The fluorescence assay is principally based on the phenomenon of target-mediated fluorescence enhancement (on) or fluorescence burst (off). It utilizes alterations in fluorescence signals generated by nanozymes catalyzed reactions to detect antioxidant capacity. In comparison to colorimetric methods, fluorescence methods exhibit a relative advantage in terms of high sensitivity [111]. Chen et al. have designed a novel single-atom copper nano-enzyme with dual enzyme mimetic activity of ascorbate OXD and POD [112]. The nanozyme oxidized the substrate AA, generating an oxidated fluorescent product that fluoresced at an excitation light of 350 nm, with the peak wavelength of its emitted light located at 425 nm (see Figure 4D). A fluorescent AA assay was developed and used to accurately determine AA and TAC in real samples (fruits, beverages, and vitamin C tablets) with high selectivity and a broader detection range. The Cu-N/C nanozyme possessed a uniform elemental distribution and exhibited higher catalytic efficiency and superior stability than natural enzymes [112]. Ni et al. have developed a fluorescence assay for the detection of antioxidant capacity using photo responsive BSA-AuNCs with OXD simulation activity for the first time. Thiamine was used as a substrate, oxidized under the catalysis of gold nanoclusters. The oxidized sulfur pigments fluoresced at the excitation light of 370 nm, with the central emission peak at 440 nm. However, the presence of antioxidants inhibited the oxidation of thiamine as shown in Figure 4E. This led to a reduction in the fluorescence signal, which enabled the quantitative detection of antioxidants. The antioxidants and TAC of vitamin C tablets, as well as some commercial fruit juices, were successfully detected, showing good applicability and reproducibility [95]. This fluorometric assay had significant advantages, such as mild detection conditions, short time, high sensitivity, etc., which can broaden the application of light-responsive nanozymes and provide a new idea for fluorometric determination. Wang et al. have investigated a fluorescent nanozymes (Cu-BDC-NH_2_) with CC oxidase activity. This nanozyme combined the dual functions of CC recognition and signal output, enabling CC detection without the addition of other color developers. In this fluorescence method, a porous organic polymer of porphyrin (FePPOP-1) was used as a fluorescent substrate. Its excitation and emission wavelengths were 330 nm and 420 nm, respectively. When reacted with CC, it catalyzed the oxidation of CC to form quinones or polymers with strong electron-absorbing capacity. It led to a significant burst change in the fluorescence intensity of FePPOP-1 at 420 nm, which quantified the concentration of CC. The detection limit was calculated to be 0.997 μmol/L, which met the need for high sensitivity detection and did not produce effective fluorescence burst with other antioxidants. This study demonstrated the advantages and potential of Cu-BDC-NH_2_ nanozyme in the field of antioxidant detection [113]. Galal et al. have introduced synchronous fluorescence spectroscopy for the simultaneous detection of curcumin and resveratrol (two natural antioxidants in plasma). By varying the value of Δ*λ* (spectral shift) in the synchrotron fluorescence mode, they have investigated the fluorescence properties of the fluorescent substance curcumin and resveratrol and finally determined the optimal value of Δ*λ* to be 80 nm. At this Δ*λ* value, the fluorescence intensities of resveratrol and curcumin reached their maximum at 304 nm and 443 nm, respectively, allowing them to be detected simultaneously without interfering with each other. The method was highly selective and capable of accurately determining target compounds in complex biological matrices without interference from plasma components. In comparison to conventional high performance liquid chromatography methods, the proposed method was more environmentally friendly and offered high accuracy and a low LOD [114]. Huang et al. have developed a novel fluorescence detection method based on a fluorescent nanocomposite material with PPO activity (Pdots@AMP-Cu) for the detection of DA, as shown in Figure 4F. In the experiment, Pdots@AMP-Cu was used as a fluorescent substance with excitation wavelength of 428 nm and emission wavelength of 668 nm, respectively. Pdots@AMP-Cu featured significant PPO catalytic activity and high stability and can catalyze 2,4-DP and 4-AP reactions in 2-(n-morpholino) ethanesulfonic acid buffer solution at pH 7. Figure 4G illustrates chemical products with distinct absorption peaks. After reacting with DA, the fluorescence was effectively quenched due to electron transfer, thus enabling the detection of DA. These nanozymes can be used for clinical diagnosis of neurological syndromes, such as schizophrenia, Parkinson’s syndrome, and Huntington’s disease. The DA in human serum samples was detected by the standard addition method, obtaining a recovery value of 97% to 105% and a relative standard deviation of no more than 4.1%, demonstrating the accuracy and reproducibility of this method in the detection of actual samples [115]. Ye et al. have synthesized zeolitic imidazolate framework-67 (ZIF-67) nanozymes by a three-step process [116]. ZIF-67 nanozymes had excellent oxidase activity and peroxidase activity, which were used to construct a highly sensitive fluorescent assay for antioxidant properties. Currently, the method enabled TA assay for nine different food products, which is expected to be used for real-time monitoring of food production safety.

#### 3.1.3. Electrochemical Method for Antioxidants Determination

**Figure 5:**
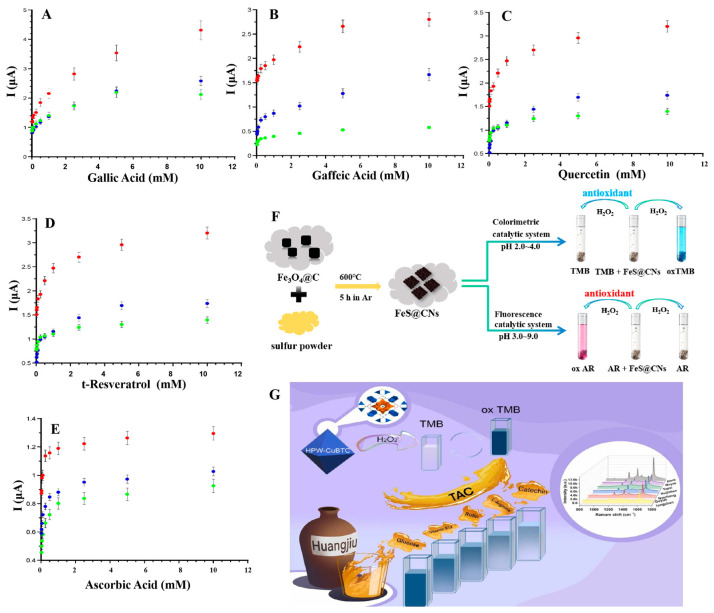
(**A**–**E**) Modified SPE sensors including OHT-069 (red circle), OHT-102 (blue circle), and OHT-000 (green circle) for antioxidant detection [118]. (**F**) Detection of H_2_O_2_ and antioxidant capacity of FeS@CNs nanozymes. (**G**) Rapid trace detection of TAC in yellow wine [121].

Electrochemical methods typically employ a target recognition unit that initially reacts with an analyte and subsequently transduces the resulting recognition event into a detectable electrical signal. Then, this electrical signal is correlated with the concentration of the analyte, enabling qualitative or quantitative detection. Electrochemical sensing strategies have attracted great attention in antioxidant capacity detection due to their rapid, sensitive, and accurate analyses. However, nanozymes are not easy to form effective electrocatalytic interfaces and can be interfered by oxidizable substances, resulting in fewer reports of nanozymes-based electrochemical assays [117]. Cristina et al. have developed a disposable electrochemical sensor based on the biomimetic properties of nano-cerium particles (CeNPs) [118]. CeNPs nanozymes can sensitively detect TAC in samples, ensuring the safety of wine or other beverages. In the experiment, an oxidation-reduction reaction occurred on the surface of the electrode modified by CeNPs, resulting in a change in current. Subsequently, cyclic voltammetry was employed, utilizing a 1.1 mmol/L solution of K_3_[Fe (CN)_6_] as a probe, to scan for alterations in the current, resulting in the emergence of peaks. A range of common antioxidants in wine was successfully tested by this method [118], including gallic acid (GA), caffeic acid (CA), quercetin (Q), and t-resveratrol (t-R) as shown in Figure 5A–E. This single-use sensor provided a simpler, cost-effective, and portable method without secondary reagents compared to traditional spectrophotometric methods. Similarly, David et al. have developed a gold nano-enzymatic electrochemical sensor with POD-like catalytic activity for the determination of antioxidants and TAC of water-soluble extracts. The electrode, modified with AuNPs, was able to detect the presence of H₂O₂ by generating an oxidation peak. Herein, the antioxidant can react with H₂O₂ thus affecting the current at the electrode. Differential pulse voltammetry was employed to assess the TAC of the extracts. The electrochemical indices, as a new quantitative method, were introduced for assessing the ‘total natural antioxidant’ or ‘total polyphenol’ content of a sample, which could be used to assess TAC of multiple samples in a short period of time. This experiment accurately assessed TAC of extracts of Sea buckthorn and Narrow-leaved Lavender, providing a new method for the assessment of TAC of plant extracts [119]. Zhang et al. have synthesized manganese dioxide nanosheets (MnO_2_ NSs) with simulated activity of catechol OXD by chemical precipitation method for the ultra-sensitive and selective electrochemical detection of DA. DA was catalytically oxidated to DA quinone on the modified electrode, which underwent electrochemical reduction at the electrode. In this process, two protons and two electrons were transferred, and by measuring the change in current at a specific potential by amperometry, the DA concentration could be quantitatively analyzed. In the presence of oxidizing substances (e.g., AA, uric acid (UA) and Cys in biological systems that may coexist with DA, there was no significant interference, demonstrating good selectivity [117]. Liu et al. have developed single-atom nanozymes with superior OXD activity for the simultaneous analysis of DA and UA in biofluids. Using Co-NNC as the active center, the nanozyme catalyzed redox reactions of DA and UA to generate electrical signals. Alterations in the electrical signal were gauged using differential pulse voltammetry and cyclic voltammetry. The experiments also employed polyvinyl alcohol hydrogel as a sweat collector in conjunction with Ppy-Co-NNC/SPCE, thereby facilitating the real-time detection of UA in sweat and the simultaneous detection of UA and DA in urine. The results demonstrated an excellent degree of selectivity, reproducibility, and stability. This novel, non-invasive biofluidic detection strategy holds important implications for personalized medical testing [120].

#### 3.1.4. Other Methods for Antioxidants Determination

There are also innovative approaches, such as the surface enhanced Raman scattering (SERS) technique, which enables highly sensitive detection of very low concentrations of antioxidants through the interaction of nanozymes with Raman-active substrates [122]. Shang et al. have developed a polyoxometalate cluster nanozymes with POD activity and have constructed a novel TAC sensing platform for the rapid and highly sensitive detection of TAC in yellow wine [121]. The HPW-CuBTC material, mixed with methyl blue molecules, can be excited by a 633 nm laser line, generating Raman scattering signals. Because of the antioxidant effect of catechin, it competed with TMB for the catalytic site of HPW-CuBTC. This reduced the generation of ox-TMB, leading to changes in Raman scattering signals and thus quantitatively assessing TAC in yellow wine samples, as shown in Figure 5G. In the study, not only was the local surface plasmon resonance enhancement mechanism used, but the charge transfer mechanism was also introduced, with the synergistic effect of the two significantly enhancing the SERS signal [121]. Similarly, Dong et al. have employed enhanced luminol chemiluminescence and have used CoMoO_4_ nanorods as a catalyst for chemiluminescence for the first time. By catalyzing the decomposition of H_2_O_2_, hydroxyl radicals (OH·) and superoxide anions (O_2_^−^) were produced. These reactive oxygen species reacted with luminal to produce intermediates in the excited state. When these intermediates returned to the ground state, they emitted light, thus enhancing the chemiluminescent signal. However, DA can react with reactive oxygen species, thereby reducing their number and leading to a decrease in luminescence intensity. Therefore, the DA in serum samples was successfully measured by this way, showing satisfactory recoveries of 97.2–104.3%, highlighting the promising application of this method in the detection of TAC [123]. In recent years, dual-mode sensing strategies [124,125,126,127,128] have been widely used to further enhance the comprehensiveness, reliability, and specificity of detection. For example, Song et al. have investigated embedded FeS@CNs nanozymes particles, which could effectively achieve the encapsulation of FeS nanozymes [125]. These particles exhibited good catalytic activity over a wide pH range and possessed both colorimetric and fluorescent dual sensing detection, as shown in Figure 5F. By creating different recognition channels, various target objects can be distinguished and the specificity and reliability of the sensing platform can be improved. These innovative methods not only improved the accuracy, sensitivity, and specificity of detection, but also provided diversified options and long-term prospects for the assessment of antioxidant capacity.

Table 1, Table 2, Table 3 and Table 4 summarize the parameters of antioxidant detection based on nanozymes using different enzyme activities and detection methods in the recent reported works.

**Table 1:** POD-like activity for antioxidant detection.

Enzyme	Detection Method	Detection Object	Detection Range (μmol/L)	LOD (μmol/L)	Reference
POD	-	AA	8~64	0.77	[90]
POD	colorimetry	AA, Cys, GSH	0.01~50,000	Fe-based nanozyme: AA (0.00517), GSH (0.00628) Cu-based nanozyme: AA (0.00377), Cys (0.00340) Zn-based nanozyme: AA (0.0063), GSH (0.00712)	[89]
POD	colorimetry	AA, Cys	AA: 0.5~120, Cys: 0.1~20	AA: 0.15, Cys: 0.06	[129]
POD	colorimetry	AA, GA, CA	AA: 0.5~50, GA: 40~600, CA: 400~1800	AA: 0.23, GA: 0.11, CA: 0.20	[107]
POD	colorimetry	Vitamin C, GSH, CYS, etc.	Vitamin C: 2~32, GSH: 4~20, Cys: 0~16, CA: 1~20, GA: 2~12	Vitamin C: 0.158, GSH: 124, CYS: 116.3, CA: 0.259, GA: 0.1885	[126]
POD	colorimetry	TAC	absorptiometry: 0~60, fluorescence spectrophotometry: 0~60	absorptiometry: 1.3, fluorescence spectrophotometry: 0.35	[124]
POD	colorimetry	H_2_O_2_, AA, ferulic acid (FA), tannin acid (TA), GA	H_2_O_2_: 5 × 10^4^~4 × 10^6^, AA: 10~80, FA: 10~100, TA: 5~60, GA: 5~40	H_2_O_2_: 16.0, AA: 8.7, FA: 8.3, TA: 2.7, GA: 2.4	[130]
POD	colorimetry	glucose	0.025~0.5	1.5	[131]
POD	colorimetry	AA	300~900	59.4	[110]
POD	colorimetry, fluorimetry	H_2_O_2_	colorimetry: 1~70, fluorimetry: 5~250	colorimetry: 0.78, fluorimetry: 0.86	[125]
POD	colorimetry	AA	2~120	0.41	[132]
POD	colorimetry	AA	1 × 10^4^~4.5 × 10^4^	6130.0	[133]
POD	colorimetry	TAC	5~40	1.40	[134]
POD	colorimetry	AA, 2,4-DP, adrenaline	AA: 0~25, 2,4-DP: 3.1~613.5, adrenaline: 1.09~272.93	AA: 0.29, 2,4-DP: 0.76, adrenaline: 0.7	[101]
POD	fluorimetry	H_2_O_2_, AA, L-Cys, etc.	H_2_O_2_: 0~4000, L-Cys: 0~4000, GSH: 0~1000, etc.	H_2_O_2_: 29.0, AA: 4.2 (spectroscopy), L-Cys: 680.0, GSH: 76.0, AA: 68.0 (fluorimetry)	[135]
POD	colorimetry	H_2_O_2_, glucose, AA	H_2_O_2_: 20~200, glucose: 10~100, AA: 8~80	H_2_O_2_: 15, glucose: 10, AA: 8	[136]
POD	colorimetry, fluorimetry	H_2_O_2_	0~60	colorimetry: 1.3, fluorimetry: 0.35	[124]
POD	colorimetry	AA, H_2_O_2_	AA: 1.0~20.0, H_2_O_2_: 100~1000	AA: 0.94, H_2_O_2_: 45.3	[137]
POD	colorimetry	AA, Cys, GSH	AA: 1~80, Cys: 1~80, GSH: 1~80	AA: 0.14, Cys: 0.18, GSH: 0.21	[108]
POD	colorimetry	H_2_O_2_, glucose, AA, etc.	H_2_O_2_: 20~1000, glucose: 15~500, AA: 1~55, etc.	H_2_O_2_: 6.5, glucose: 3, AA: 0.35, GA: 0.75, TA: 0.048	[138]
POD	colorimetry	AA, GSH, TA, etc.	0.01~50,000	0.01	[89]
POD	colorimetry	GLY, GSH, CA, etc.	0.1~10	0.1	[139]
POD	colorimetry	AA, Cys	0.5~120	0.06 (Cys), 0.15 (AA)	[129]
POD	colorimetry	GSH, AA, Cys, etc.	0.01~50	10	[105]
POD	colorimetry	Cys, UA, polyphenols, etc.	0.01~10.0	0.000116(AA), 0.000112 (Cys), 0.000143 (DA), etc.	[127]
POD	colorimetry	AA	10.0~125.0	0.406	[140]
POD	colorimetry	hydroperoxide	10~10,000	1.55	[141]

-: not illustrated.

**Table 2:** OXD-like activity for antioxidant detection.

Enzyme	Detection Method	Detection Object	Detection Range (μmol/L)	LOD (μmol/L)	Reference
OXD	colorimetry	TAC	1.25~10	0.00825	[104]
OXD	colorimetry	GSH, AA, Cys, etc.	-	0.01	[105]
OXD	colorimetry	AA, GSH, Cys	AA: 1~30, GSH: 1~30, Cys: 2~10	AA: 1.53, GSH: 2.00, Cys: 0.97	[94]
OXD	colorimetry	TAC	1~30	1.17	[93]
OXD	colorimetry	TH, AA, GSH	TH = 7: 0.1~60, TH > 7: 0.005~1, AA: 3~50, GSH: 1~40, etc.	-	[95]
OXD	colorimetry	AA, 2,4-DP, adrenaline	AA: 0~25, 2,4-DP: 3.1~122.7 and 122.7~613.5, adrenaline: 1.09~109.2 and 109.2~272.93	AA: 0.29, 2,4-DP: 0.76, adrenaline: 0.70	[101]
OXD	colorimetry, fluorimetry	GA	colorimetry: 0~60, fluorimetry: 0~60	colorimetry: 1.3, fluorimetry: 0.35	[101]
OXD	colorimetry	TAC	-	-	[96]
OXD	colorimetry	AA, GSH, homocysteine	AA: 1~90, GSH, homocysteine: 3~70, 2.5~50	AA: 0.2, GSH and homocysteine: 0.8 and 0.9	[142]
OXD	colorimetry	AA, Cys, GSH	AA: 3.0~25, Cys: 3.0~33, GSH: 3.0~35	AA: 0.04, Cys: 0.047, GSH: 0.067	[143]
OXD	colorimetry	GA, 4-hydroxycinnamic acid, anthocyanidin, etc.	5~100	5	[144]
OXD	electrochemical method	phenol	0.01~0.2	0.00294	[145]

-: not illustrated.

**Table 3:** Laccase-like activity for antioxidant detection.

Enzyme	Detection Method	Detection Object	Detection Range (μmol/L)	LOD (μmol/L)	Reference
laccase	colorimetry	CC	5.0~70.0	2	[146]
laccase	electrochemical method	CC	0.036~2.5	0.032	[147]
laccase	electrochemical method	polyphenol compounds	1~250	0.83	[148]
laccase	electrochemical method	total Phenolic Compounds	0.1~500	0.05	[149]
laccase	electrochemical method	phenolic compounds	0.1~500	0.03	[150]
laccase	electrochemical method	polyphenol	1~500	0.156 (P-guaiacol)	[151]
laccase	colorimetry	CC, HQ	1~1000 (CC), HQ: 0.05~100	0.35 (CC), HQ: 0.04	[102]
laccase	electrochemical method	2-aminophenol, catechol, etc.	50~1000	-	[152]
laccase	colorimetry	2,4-dichlorophenol, phenol, CC, etc.	0.1~100	0.033	[98]
laccase	electrochemical method	polyphenol	0.01~10	0.081	[153]
laccase	colorimetry	2,4-dichlorophenol, phenol, CC, etc.	0.1~2000	34,000 (2,4-dichlorophenol)	[154]
laccase	fluorimetry	GA	-	7.4	[155]
laccase	electrochemical method	CC	3.0~15	0.91	[156]
laccase	electrochemical method, optics method	polyphenol, including GA, CA, etc.	0.1~100 or higher	0.0001~ 0.7	[157]
laccase	electrochemical method	RA	0.91~12.1	0.233	[158]
laccase	colorimetry, combined with smart phone platform	AA	AA: 0~25, 2,4-dichlorophenol: 3.1~613.5, adrenaline 1.09~272.93	AA: 0.29, 2,4-dichlorophenol: 0.76, Adrenaline: 0.70	[101]
laccase	fluorimetry, colorimetry	TAC, AA	AA: 10~130, TAC: 10~100	AA: 0.70, TAC: 0.30	[112]
laccase	fluorimetry	catechins, epicatechins and polyphenol, etc.	1 ng/mL~100 mg/mL	1 ng/mL	[159]
laccase	colorimetry	CA, GSH, Trolox	CA: 0.01~130, Trolox: 0.01~180, GSH: 1~100	CA: 0.00483, Trolox: 0.00739, GSH: 0.00889	[91]

-: not illustrated.

**Table 4:** Multienzyme activity for antioxidant detection.

Enzyme	Detection Method	Detection Object	Detection Range (μmol/L)	LOD (μmol/L)	Reference
OXD, catalase, laccase	colorimetry	TAC, phenol compound	AA: 0~25; 2,4-DP: 3.1~613.5; adrenaline: 1.09~272.93	AA: 0.29; 2,4-DP: 0.76; adrenaline: 0.70	[101]
OXD, POD	colorimetry	DA, GSH, AA, etc.	0.01~0.25	DA: 0.00826, AA: 0.00542, GSH: 0.00289, Cys: 0.00624	[160]

### 3.2. Nanozymes-Based Antioxidant Detection Device

Antioxidants are considered essential compounds for monitoring human health. With the increasing concern about antioxidants, detecting antioxidant levels more accurately and rapidly has become a new direction for more researchers. In recent years, the combination of nanozymes and sensor arrays, paper-based devices, and microfluidic devices [145] for the detection of antioxidants has been gradually increasing, providing new ideas for providing rapid detection in the field.

#### 3.2.1. Sensor Array Devices

**Figure 6:**
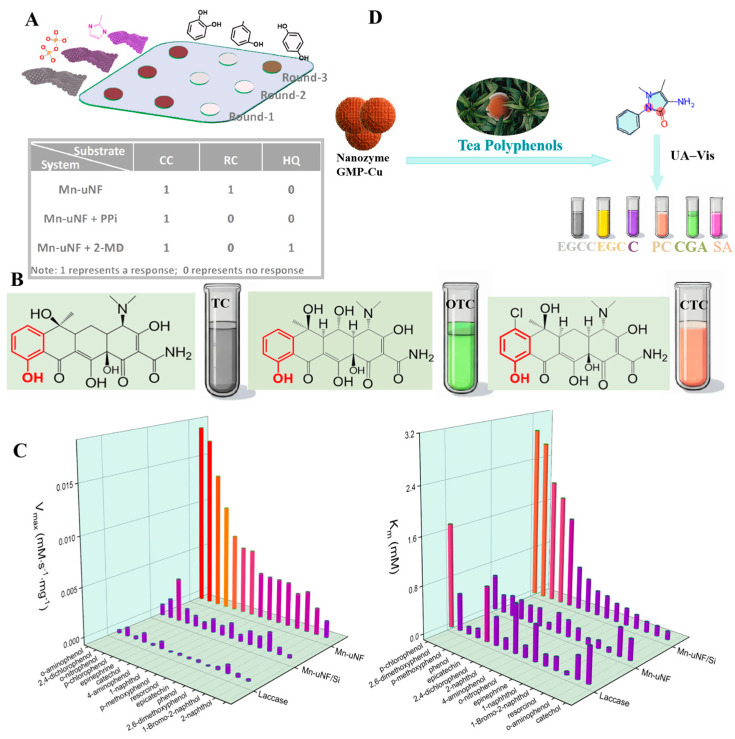
(**A**) Cross-reactive sensor array [161]. (**B**) Chemical structures of tetracyclines and results of colorimetric reactions. (**C**) Catalytic activities of laccase, Mn-uNF/Si, and Mn-uNF for the oxidation of various phenolic compounds (*V_max_* and *K_m_*) [161]. (**D**) Different reactions of nanozymes with polyphenol OXD activity to tea polyphenols.

**Figure 7:**
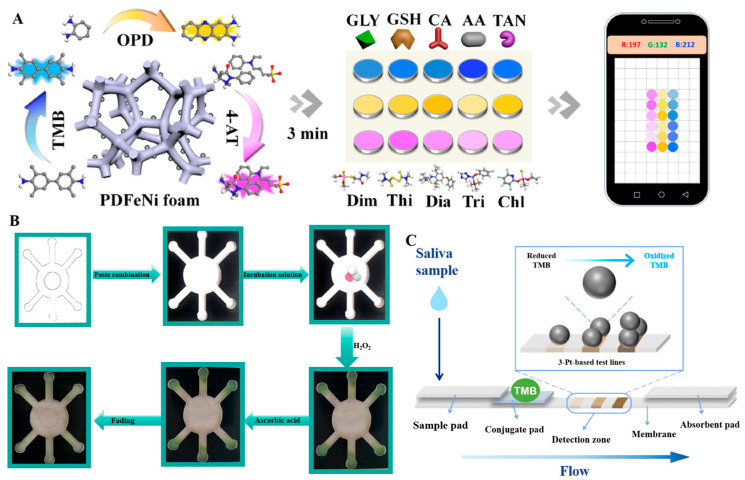
(**A**) Accelerated and precise identification of antioxidants and pesticides using a smartphone-based colorimetric sensor array [139]. (**B**) Schematic of the process of determining apparent AA content on a paper-based microfluidic chip [140]. (**C**) Design of a platinum nanozyme-based lateral flow assay [174].

Sensor arrays are composed of multiple sensing elements, and different elements will selectively interact with the target to generate special “fingerprints”, thus enabling the identification of various analytes. It is an outstanding advantage for the identification of mixed samples compared with a single sensor that can only detect one physical or chemical quantity. Wu et al. have designed a colorimetric sensor array consisting of three sensing units with two-dimensional ultrathin manganese dioxide nanofilms (Mn-uNF) with laccase-like functionality to identify HQ, resorcinol (RC), and CC [144]. As shown in Figure 6A, the recognition of isomers was achieved by the difference in the color development reaction of the three isomers with the three sensing units. The Mn-uNF-based sensing platform possessed good sensitivity and stability. The array was capable of detecting RC down to 2.7 µmol/L and recognizing tetracyclines (TCs) containing phenolic groups. The Mn-uNF-based sensing platform provided good sensitivity and stability. The sensor enabled the recognition of tetracyclines containing phenolic moieties using laccase mimics. In Figure 6B, there is a clear difference between the colorimetric responses of TC, oxytetracycline (OTC), and chlortetracycline (CTC) with this sensor array, which makes the recognition of TCs more specific and intuitive and breaks through the limitations of the traditional cross-reactive sensor arrays. In addition, the designed Mn-uNF had high catalytic efficiency and good affinity. In Figure 6C, the *V_max_* of Mn-uNF for o-aminophenol was 21.1 times higher than that of laccase, and its *K_m_* (0.301 mmol/L) was lower than that of laccase (0.493 mmol/L), indicating a higher affinity for the substrate. Yang et al. have constructed a sensing array with 15 sensing units using nanozymes (GMP-Cu) [144], and processed the data using principal component analysis. Different tea polyphenols produced unique changes in the sensing units, enabling differentiate between different polyphenols. The differences among various tea polyphenols in green tea were significant. In Figure 6D, the reactions of nanozymes can be utilized to recognize and differentiate the content and type of these tea polyphenols. The detection limit of this sensing array was 5 µmol/L, and it was highly accurate in the presence of interferences such as vitamin C, glucose, K^+^, Mn^2+^, etc. Therefore, this system is suitable for accurate antioxidant testing of tea samples in production. Qin et al. chose Co-based nanozymes as the sensing element, constructed a colorimetric sensor array using the 3D lattice structure of the nanozymes [89], and recorded multiple response patterns (2 nanozymes × 7 antioxidants × 5 replicates) for linear discriminant analysis. The optimal pH, antioxidant time, reaction time, and reaction temperature conditions of the nanozymes were also optimized. In addition to this, all three nanozymes in the study were able to detect antioxidants down to 10 nmol/L. The sensor array was able to recognize seven antioxidants even at low concentrations, with the metal-based nanozymes -based sensor array providing a good method for antioxidant detection. Liu et al. have investigated the Co-N-C nanozymes prepared with POD activity and showed different colorimetric responses to oxidized TMB at pH = 3.8 and pH = 4.6 [105]. Based on this, a sensor array with two sensing units was developed, and the data were processed using a chemometric method such as linear discriminant analysis (LDA). The sensor array was able to recognize seven antioxidants at a concentration of 10 nmol/L with 100% accuracy. This study provided a good strategy for recognizing multiple antioxidants. Some of the sensing arrays have the capability to detect other substances along with antioxidants. For example, Tian et al. have developed a three-channel colorimetric sensing array based on PDFeNi foam, which incorporated a smartphone for rapid signal reading to detect antioxidants and pesticides, as shown in Figure 7A. The PDFeNi foam in this sensing array was a nanomaterial with POD enzyme activity, prepared through polydopamine modification of the FeNi foam.

#### 3.2.2. Paper-Based Devices

Paper-based detection is a paper-based technology that reduces the cost of manufacturing detection devices, simplifies the fabrication process, and can be used to build various types of devices, including sensors [162,163,164,165,166,167,168], actuators [169,170,171,172], etc. Compared with traditional detection devices, it has the advantages of low cost and easy operation, and can better adapt to the needs of anytime, anywhere detection. Anna et al. have designed a multiplexed colorimetric method for the determination of antioxidants in wine by paper [173]. This analytical method required only a low-cost x-y plotter, markers, and paper, and was environmentally friendly, cost-effective, instrument-free, and rapid. Traditional paper-based methods are no longer sufficient to meet the demand for highly sensitive assays, so combining nanozymes with paper-based devices and using them for sensitive antioxidant assays has become a new development in paper-based devices. K.V. et al. have observed for the first time the POD mimetic performance of chitosan against H_2_O_2_ and TMB by deploying a paper-based colorimetric sensor, which was of great significance in the development of biosensors. The sensor was made of chromatographic paper that was wax-printed, making the sensing points hydrophilic and the boundaries hydrophobic. The detection limit of the chitosan method was 1.55 µmol/L with high sensitivity, allowing rapid detection in less than 10 min, which could be read by a smartphone. It was portable and responsive for on-site testing. Guan et al. have constructed a paper-based microarray for visual detection by embedding AuNPs on paper sheets and utilizing the POD-like activity of the nano-enzymes for on-site and quantitative detection of AA [140], the principle of the established paper-based platform. In Figure 7B, a mixed solution containing nanozymes and 3-ethylbenzothiazole-6-sulfonic acid was added to the intermediate area, with a certain amount of H_2_O_2_ added equally to the detection area. Finally, the mixed solution penetrated the detection area to undergo a color development reaction. The detection limit for antioxidants was as low as 0.406 μmol/L with high sensitivity, which provided a new idea for designing highly active nanozymes for on-site detection of dietary components. Anna et al. have developed a new instantaneous detection technique for TAC using the POD-like nature of 5 nmol/L platinum nanoparticles (PtNPs) [174]. PtNPs were combined with a colorimetric paper device for the detection of antioxidants. The device consisted of a series of pads (sample, coupling, and absorbent pads), as shown in Figure 7C. The saliva sample contained physiological antioxidants, and the detection zone consisted of three strips with different levels of nanozymes. These nanozymes catalyzed reaction, eventually presenting a certain number of bands, which allowed for the measurement of high, medium, and low levels of antioxidants.

#### 3.2.3. Microfluidic Devices

Microfluidic devices use micropipettes to handle or manipulate tiny fluids, allowing for a range of microfluidic processing and micromanipulation tasks that are difficult to accomplish with conventional methods. Whereas conventional assays typically require large sample volumes to perform the assay, microfluidic devices can reduce the amount of sample required and allow for high-throughput implementation. Lee et al. have developed a customized spectrometer system with an LED light source and a dual chamber microfluidic system [175]. A simplified analytical model and a complex finite element model were built, borrowing from reaction engineering and fluid dynamics. The system enabled the tracking of the progress of the reaction and detected antioxidant activity of a substance in a physiologically relevant manner. This microfluidic device featured the ability to monitor the effect of the chemical reaction in real time, saving detection time to achieve the purpose of rapid and batch detection. Microfluidic devices are the latest advances in microfluidic technology, which not only can greatly reduce the cost of the assay, but also can be more sensitive and faster. Guan et al. have combined paper-based technology with microfluidic technology to construct a paper-based microfluidic chip based on AuNPs nanozymes, integrating the data analysis with smartphones [140]. This method was strong in anti-interference ability and simple in operation, which provided a new idea for the application of highly active nanozymes in microfluidic devices. Wu et al. have developed a novel microfluidic sensor combining nano-enzymes and microfluidic chips to detect the TAC with a detection limit of 33.4 µmol/L and high sensitivity [176]. The device consisted of two layers of plastic laminates with glass fiber paper containing nanozymes in the upper layer and filter paper immobilized with TMB in the lower layer. The method reduced sample pre-treatment and allowed the assay to be completed in less than 15 min, saving a great deal of time. The method did not require expensive specialized equipment and simplified the operation process, which can be applied to the immediate detection in the field and provided a new idea for the design of testing equipment for the detection of TAC.

## 4. Summary and Future Perspectives

In the context of rapid development of nanotechnology, the use of nanozymes and their associated devices for the detection of antioxidants has become an inevitable trend. In this review, different enzyme-like activities for antioxidant detection are categorized according to the catalytic properties of nanozymes. Meanwhile, some nanozymes-based sensing strategies, including colorimetric, fluorescence, and electrochemical methods, were introduced. A variety of nanozymes-based antioxidant detection devices, including sensor arrays, paper-based devices, and microfluidic devices, was reviewed.

At present, the use of nanozymes for antioxidant detection is still challenging. The design cycle of novel nanozymes is long and expensive. The construction and improvement of sensing devices are costly. Additionally, due to the complication of the catalytic reaction process of nanozymes, some mechanisms are still unclear. Materials used for nanozymes preparation receive great attention, and a large number of non-metallic and metallic materials are expected to be developed and used for nanozymes production. The toxicity of nanozymes cannot be ignored in the process of testing the antioxidant. This is a continuous improvement process, which may take a long time to develop from the foundation to the completion. Applying the novel nanozyme-based devices to detect antioxidant and realizing their commercialization is an inevitable trend. Good quality (good sensing performance) and detection efficiency are important factors in the process of commercializing and marketing of antioxidant detection devices. Therefore, in the future, advanced preparation processes (such as 3D printing, printed electronics, or sol-gel technology, etc.) should be used to further improve the accuracy and reproducibility of sensing devices. In addition, self-driven microfluidic chips and humanized integrated software can also be designed to reduce the difficulty of operation and simplify the operation process.

The rapid development of artificial intelligence (AI) is driving the detection field into a new era of intelligence and automation. Machine learning (ML) is a subset of AI. ML, especially models built on artificial neural systems such as feed-forward neural networks and recurrent neural networks, enables efficient learning through synergistic effects. ML shows great potential in the field of materials development. ML helps guide the development of next-generation nanozymes through intelligent enzyme design, effectively avoiding wasted time and resources. At present, AI techniques, including ML, are being used for enzyme engineering predictions, such as predicting enzyme function, catalytic sites, enzyme activity, and unknown reactions. This technology is conducive to promoting the development of nanozymes for efficient detection of antioxidants. At the same time, AI-driven detection technology will make antioxidant detection more automated and accurate. AI algorithms are able to identify and read the most relevant features of a particular phenomenon from a data model, which in turn enhances the data analysis of nanozymes based sensors. For example, during a colorimetric assay, ML can interpret and analyze chromogenic changes for accurate determination of experimental results. This provides new perspectives and insights to advance fast, efficient, and accurate assays.

In summary, this review clarifies the importance and superiority of nanozymes in antioxidant detection. The detection equipment based on the AI will make a significant contribution to achieving antioxidant testing more successfully and economically.

## Data Availability

Not applicable.
